# A new strain group of common carp: The genetic differences and admixture events between *Cyprinus carpio* breeds

**DOI:** 10.1002/ece3.6286

**Published:** 2020-04-27

**Authors:** Artem V. Nedoluzhko, Natalia V. Slobodova, Fedor Sharko, Gulmira M. Shalgimbayeva, Svetlana V. Tsygankova, Eugenia S. Boulygina, Zsigmond Jeney, Van Q. Nguyen, Thế T. Pham, Đức T. Nguyen, Alexander A. Volkov, Jorge M.O. Fernandes, Sergey M. Rastorguev

**Affiliations:** ^1^ Faculty of Biosciences and Aquaculture Nord University Bodø Norway; ^2^ National Research Center “Kurchatov Institute” Moscow Russia; ^3^ Institute of Bioengineering Research Center of Biotechnology of the Russian Academy of Sciences Moscow Russia; ^4^ Kazakh Research Institute of Fisheries Almaty Republic of Kazakhstan; ^5^ National Agricultural Research and Innovation Center Research Institute for Fisheries and Aquaculture (HAKI) Szarvas Hungary; ^6^ Institute of Marine Environment and Resources Vietnam Academy of Science and Technology Hanoi Vietnam; ^7^ Graduate University of Science and Technology Hanoi Vietnam; ^8^ Russian Federal Research Institute of Fisheries and Oceanography Moscow Russia

**Keywords:** admixture, amur carp subspecies, carp strain groups, *Cyprinus carpio*, domestic strains, RAD sequencing

## Abstract

Common carp (*Cyprinus carpio*) has an outstanding economic importance in freshwater aquaculture due to its high adaptive capacity to both food and environment. In fact, it is the third most farmed fish species worldwide according to the Food and Agriculture Organization. More than four million tons of common carp are produced annually in aquaculture, and more than a hundred thousand tons are caught from the wild. Historically, the common carp was also the first fish species to be domesticated in ancient China, and now, there is a huge variety of domestic carp strains worldwide. In the present study, we used double digestion restriction site‐associated DNA sequencing to genotype several European common carp strains and showed that they are divided into two distinct groups. One of them includes central European common carp strains as well as Ponto–Caspian wild common carp populations, whereas the other group contains several common carp strains that originated in the Soviet Union, mostly as cold‐resistant strains. We believe that breeding with wild Amur carp and subsequent selection of the hybrids for resistance to adverse environmental conditions was the attribute of the second group. We assessed the contribution of wild Amur carp inheritance to the common carp strains and discovered discriminating genes, which differed in allele frequencies between groups. Taken together, our results improve our current understanding of the genetic variability of common carp, namely the structure of natural and artificial carp populations, and the contribution of wild carp traits to domestic strains.

## Graphical abstract

We showed that the studied domestic strains are divided into two clearly distinct groups. One of the groups has traces of genomic introgression of wild Amur carp (*Cyprinus carpio haematopterus*). We defined genes with significantly different allele frequency between the groups and conducted functional gene set analyses to estimate gene categories, enriched in the gene set that discriminates between strains.

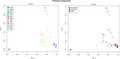

## 1 INTRODUCTION

Common carp (*Cyprinus carpio*) is a species of the Cyprinidae family, which is the largest and most diverse fish family (Nelson, 1995). Its natural habitat ranges from Western Europe to China, Korea, Japan, and Southeast Asia; from Siberia at 60°N to the Mediterranean Sea and India (Gross, Kohlmann, & Kersten, 2002). The common carp was also the first fish species to be domesticated in China, around the 5th century BC, at the same time it was being cultivated at the peak of the Roman Empire in Europe (Balon, 2006). To date, there is no consensus about the origin of common carp—some investigators suggest that it originated in the Caspian and Aral Sea regions, from where it spread in both East and West directions (Balon, 1995). Others support that the common carp has its origin in Eastern Asian, where it was domesticated and then spread to Europe during the Greco‐Roman period (Zardoya & Doadrio, 1999).

The karyotype of common carp consists of 100 chromosomes, more than in most other fish species. Because of tetraploidization, many genes in the carp genome have paralogues (Ohno, Muramoto, Christian, & Atkin, 1967). Despite the nuclear genome complexity, carp species are widely used for evolution, phylogeography, and population genetic studies because of their ecological and economic importance (Chistiakov & Voronova, 2009; Gui & Zhu, 2012; Vilizzi, 2012). As one of the most economically important fish species, its worldwide production exceeded 4 million tons in 2015, according to the Food and Agriculture Organization, of which hundred thousand tons were wild caught (FAO, 2015). The successful farming of common carp is linked to its long history of domestication. Artificial selection and crossbreeding to wild specimens has led to the creation of more than 35 domestic strains (Hulata, 1995). Hence, common carp is a suitable fish model for domestication studies of artificial trait selection and history of hybridization.

Originally, the Russian geneticist Valentin Kirpitchnikov (1908–1991) distinguished four subspecies of common carp: *C. carpio carpio* (Europe), *C. carpio aralensis* (Central Asia), *C. carpio haematopterus* (Asia), and *C. carpio viridiviolaceus* (Southeast Asia), based on morphological data (Kirpitchnikov, 1967). Subsequent genetic studies, based on mitochondrial and microsatellite DNA analyses, did not confirm a separate subspecies status of *C. c. aralensis*, because it is closely related to *C. carpio carpio* (Kohlmann, 2003; Kohlmann, Kersten, & Flajšhans, 2005; Memiş & Kohlmann, 2006). The status of the Southeast Asia common carp remains unclear (Kohlmann et al., 2005). Nowadays, *C. carpio* is usually divided into at least two distinct subspecies: Ponto–Caspian (*C. carpio carpio*) and Eastern Asian (*C. carpio haematopterus*), according to microsatellite and mitochondrial data (Kohlmann et al., 2003, 2005; Zhou, Wang, Ye, & Wu, 2003; Zhou, Wu, Wang, & Ye, 2004).

Genome sequencing of European and Asian domestic common carp strains showed that they formed two distinct groups, as a consequence of their diverse geographical habitats and domestication histories (Xu et al., 2014). However, this study did not include the additional common carp strains that had been created in the Soviet Union in the XX century (Ludannyĭ, Khrisanfova, Prizenko, Bogeruk, & Semenova, 2010). A specific feature of this group is its adaptation to cold. To reach this characteristic, domesticated strains were bred with wild Amur carp (*C. carpio haematopterus*), which inhabits the Amur River on the Russian Far East, and their offspring underwent artificial selection for low‐temperature resistance. Here, we marked this domestic group as the Northern carp strain group based on its origin, even if some of these strains are now cultivated in southern regions of Russia (e.g., Stavropol and Ukrainian common carp strains).

Restriction site‐associated DNA sequencing (RAD sequencing) is a state‐of‐the‐art approach for genotype analysis, which has the advantages of next‐generation sequencing (NGS) technology for population‐wide studies with relatively low cost (Hohenlohe et al., 2010). A few modifications of the method have been developed to date, one of them known as double digestion restriction site‐associated DNA (ddRAD) sequencing (Franchini, Monné Parera, Kautt, & Meyer, 2017) allows large‐scale sample multiplexing.

In the present study, we analyzed 68 specimens of common carp from nine different domestic strains and four wild populations using ddRAD sequencing. We showed that the studied domestic strains are divided into two clearly distinct groups. Moreover, we demonstrated that one of them has traces of genomic introgression of the wild Amur carp (*C. carpio haematopterus*). We found several genes with significantly different allele frequency between groups and conducted functional gene set analyses to estimate gene categories, enriched in the gene set that discriminates between strains.

## 2 MATERIALS AND METHODS

### 2.1 Sampling, DNA extraction, library preparation, and sequencing

The 68 individuals of thirteen domestic strains and wild populations of common carp were obtained from the Russian National Collection of Reference Genetic Materials (RNCRGM) of the Russian Federal Research Institute of Fisheries and Oceanography (VNIRO), Moscow, Russia, and from the Live carp gene bank of the Research Institute for Fisheries and Aquaculture Hungary (HAKI), Szarvas, Hungary. All samples were received as ethanol fixed clips of fin. The numbers of specimens, strain names, and their sources are shown in Table 1.

**Table 1:** Common carp specimens that were used in this study, their sources, and accession numbers

Strain/population name	PCA abbreviation	Source	Number of specimens	Strain group	NCBI accessions
Amur	Amur	VNIRO	5	Wild Northern	SAMN12827358–SAMN12827362
Angelinskii	Ange	VNIRO	5	Northern	SAMN12827363–SAMN12827367
Cherepets	Cher	VNIRO	5	Northern	SAMN12827393–SAMN12827397
Ropsha	Rops	VNIRO	5	Northern	SAMN12827403–SAMN12827407
Ukrainian	Ukra	HAKI	5	Northern	SAMN12827426–SAMN12827430
Stavropol	Stav	VNIRO	5	Northern	SAMN12827408–SAMN12827412
Czech	Czec	HAKI	5	Ponto–Caspian	SAMN12827383–SAMN12827387
Fresinet	Fres	HAKI	5	Ponto–Caspian	SAMN12827388–SAMN12827392
Poljana	Polj	HAKI	5	Ponto–Caspian	SAMN12827398–SAMN12827402
Tata	Tata	HAKI	5	Ponto–Caspian	SAMN12827413–SAMN12827417
Tisza	Tisz	HAKI	8	Wild Ponto–Caspian	SAMN12827418–SAMN12827425
Ural	Ural	VNIRO	5	Wild Ponto–Caspian	SAMN12827431–SAMN12827435
Volga	Volg	VNIRO	5	Wild Ponto–Caspian	SAMN12827446–SAMN12827450

As some strains have a different type of scaliness (scaled, linear, scattered, and nude), we used only scaled samples for uniformity and comparability with wild *C. carpio* specimens. Description of the strains, maintained at HAKI, Szarvas, Hungary, with their qualitative and quantitative characteristics, is available online at FAO (http://www.fao.org/3/y2406e/y2406e00.htm#Contents).

Genomic DNA was isolated from ethanol‐preserved fins by proteinase K digestion at 50°C for 16–20 hr, followed by purification through phenol‐chloroform extraction, ethanol precipitation, and resuspension in sterile ddH_2_O (Sambrook, Fritsch, & Maniatis, 1989).

Purified DNA was quantified using a Qubit 2.0 fluorometer (Invitrogen), and DNA integrity was assessed by agarose gel electrophoresis.

The library preparation protocol followed the general principles of the quaddRAD approach (Franchini et al., 2017). Genomic DNA was digested with *MspI* and *PstI* restriction endonucleases (NEB, Ipswich, USA) in the presence of adapters with six base pairs (bp) inner index sequences and four random bases to remove PCR duplicates. The digestion step was conducted in the presence of ligase. The libraries were then pooled in six groups of 12 libraries and amplified using primers with outer 8 bp indexes. Agarose gel size selection was used for reducing the genome fraction for further DNA sequencing. An S2 flow cell of Illumina Novaseq6000 genome analyzer (Illumina) with paired‐end reads (2 × 150 bp length) was used for ddRAD libraries sequencing.

### 2.2 Raw read processing and mapping

Raw ddRAD‐seq reads were processed with the Stacks package version 2.41 (Rochette & Catchen, 2017). The *clone_filter* module of Stacks was used for PCR duplicate removal. *Process_radtags* was used for demultiplexing the dual index reads and to remove erroneous and low‐quality reads (options: ‐c ‐q).

The obtained cleaned paired reads were mapped to the reference genome of common carp (RefSeq assembly accession: GCF_000951615.1) using Bowtie2 (Langmead, Wilks, Antonescu, & Charles, 2017) with the *very‐sensitive* parameter. The mapped data in SAM format were converted to binary (BAM) format, sorted and then indexed by Samtools v 0.1.19 (Li, 2011).

### 2.3 Genotype calling and discriminant analyses

SNP calling was conducted by Bcftools v 1.9 (Li, 2011) with maximum base quality—30 (‐‐min‐BQ parameter)—and with depth coverage information for each SNP loci as INFO tag to output in a VCF file (‐‐annotate DP parameter). This VCF file was loaded into R statistic environment (www.r‐project.org) by the vcfR package (Knaus & Grünwald, 2017). After loading, SNP data were filtered by SNP locus coverage, dropping out loci with coverage less than 10X. VCF was then converted into genlight format of the adegenet R package (Jombart & Ahmed, 2011), and the StaMPP R package was used to calculate population genetic statistics, such as Nei's distances and Fst (AMOVA‐based statistics) (Pembleton, Cogan, & Forster, 2013). To test loci for the probability of agreement with Hardy–Weinberg equilibrium, based on observed frequencies of homozygotes and heterozygotes, we used the gl.report.hwe function of dartR in R (Gruber, Unmack, Berry, & Georges, 2018). We also used adegenet for discriminant analysis (DAPC). Clustering based on dissimilarity matrix was conducted using Gdsfmt and SNPRelate R packages (Zheng et al., 2012); other genetic distance estimations and dendrogram plotting were conducted by the Ape R package (Paradis & Schliep, 2019).

Admixture analyses of wild Amur carp to domestic common carp strains were performed with the NGSAdmix software (Skotte, Korneliussen, & Albrechtsen, 2013) setting the number of clusters (‐K parameter) to two.

### 2.4 Differential gene analyses

To estimate loci with differences in allele frequency between European and Northern carp strain groups, we selected specimens of domestic strains from VCF file, filtered by coverage (the loci with more than 10X coverage as minimum in 80% specimens), and imported them to the plink2 package (Chang et al., 2015) for logistic regression association statistics analysis. Loci (*p‐*value <.05) were estimated for applicability to distinguish two *C. carpio* strain groups and then were selected for further analysis.

Genomic positions of the selected loci were intersected with gene positions, annotated using the reference genome of common carp and the bedtools software (Quinlan & Hall, 2010). These genes containing the selected polymorphisms were submitted to gene ontology (GO) analysis.

As the functional gene list analysis is only available for a restricted number of species, we converted carp gene IDs to the most appropriate model species—zebrafish because it is relatively closely related to the *C. carpio*. To define a GO category for each carp gene, their fasta sequences were compared to *D. rerio* amino acid sequences (*D. rerio* peptide database v. GRCz11) using blastx tool (Lobo, 2008). The *D. rerio* peptide IDs with the best blastx scores for the *C. carpio* genes were converted to their gene IDs and used for further GO analyses. *D. rerio* gene IDs were also converted to the corresponding human gene IDs for several functional analyses (functional gene annotation and clustering), using the *DAVID* Bioinformatics Resources 6.8 conversion Tool, NIAID/NIH (Hosack, Dennis, Sherman, Lane, & Lempicki, 2003). Functional analyses were conducted using the Panther server (http://pantherdb.org) (Mi, Muruganujan, Ebert, Huang, & Thomas, 2019).

## 3 RESULTS

We obtained two fasta files for each ddRAD library after demultiplexing, PCR duplicate trimming, and quality filtering. In total, 982,827 variable loci from the 68 specimens of 13 common carp populations (domestic strains and wild populations) were obtained after mapping and SNP calling, but 65,686 loci remained after filtering by coverage—only loci with more than 10X coverage as minimum in 80% specimens were selected. Among them, 1,819 filtered loci had more than two alleles and were therefore not used in subsequent analyses.

The Hardy–Weinberg test has shown that 3,618 (from the 65,686) loci have a deviation from equilibrium in at least one strain or wild population—about 5% of deviated loci.

**Figure 1:**
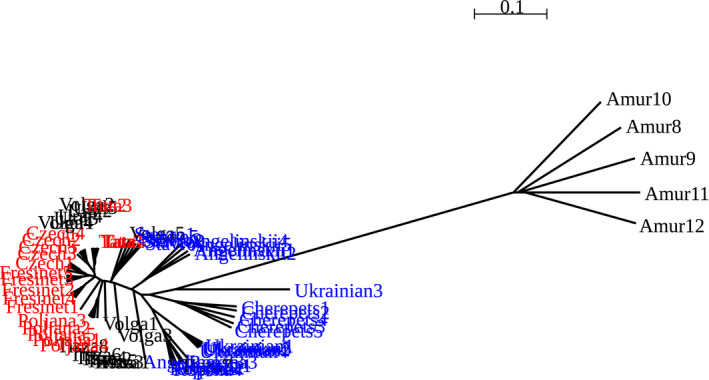
Cluster analysis of common carp performed on genome‐wide identity by state (IBS) pairwise distances. Blue font indicates specimens from the Northern strains, the European strains are shown in red, and wild individuals are indicated in black

Dissimilarity matrix‐based reconstruction revealed clearly differentiated carp strain clusters, despite small distances between specimens (Figure 1). Generally, the two main strain groups can be distinguished in the clustering: Ponto–Caspian (European) cluster contains Poljana, Czech, Fresinet, and Tata strains as well as Volga, Ural, and Tisza wild common carp populations, while the Northern (mostly, Russian) strains form a separate group of strain branches in the dendrogram. This cluster contains Angelinskii, Cherepets, Ropsha, Ukrainian, and Stavropol strains.

**Figure 2:**
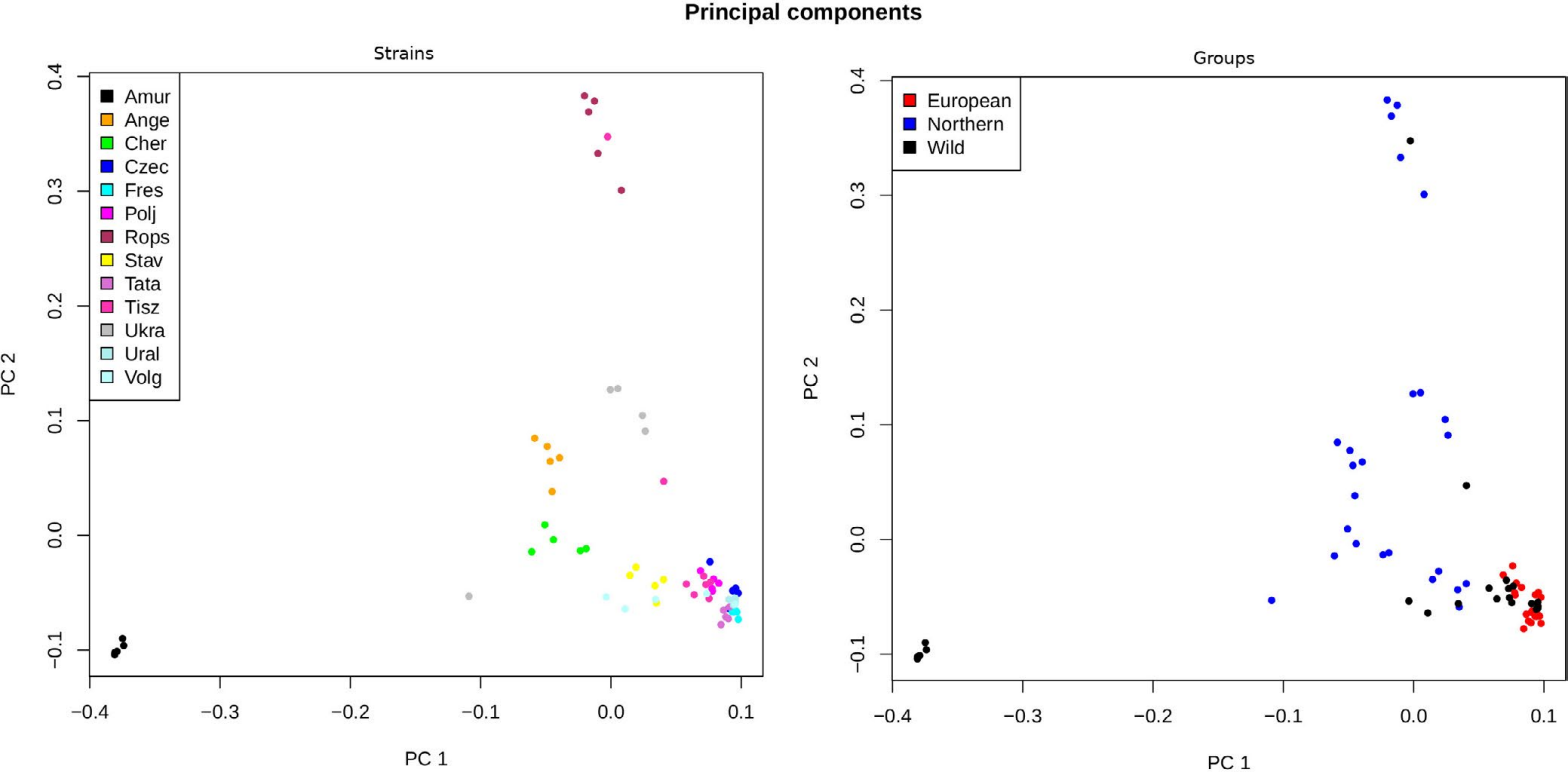
PCA plot of common carp specimens by genotype distances in (a) different populations and (b) common carp strain groups

According to distances in the dendrogram, the Ponto–Caspian (European) strains are closer to each other than the Northern strains. A similar result was obtained from the PCA analysis (Figure 2). The Ponto–Caspian (European) specimens group together, while Northern ones are scattered across the plot area. The wild Amur carp samples are the most distant from other samples of the plot, which is confirmed by the dendrogram on Figure 1.

**Figure 3:**
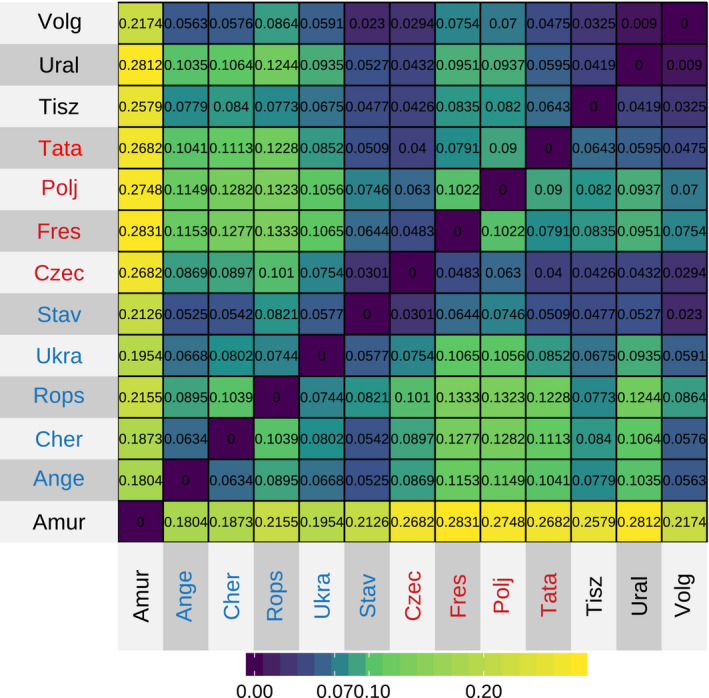
Heatmap plot of pairwise *F*st distances between each common carp strains and wild populations

Estimation of Fst distances (*p‐*value <.01) between all carp populations also shows that wild Amur carp has the longest *F*st distances from others (Figure 3). The *F*st distances between European strains are shorter, followed by the Northern strains. This observation corroborates the PCA and dendrogram reconstruction, where European strains are situated closer to each other than the Northern ones.

**Figure 4:**
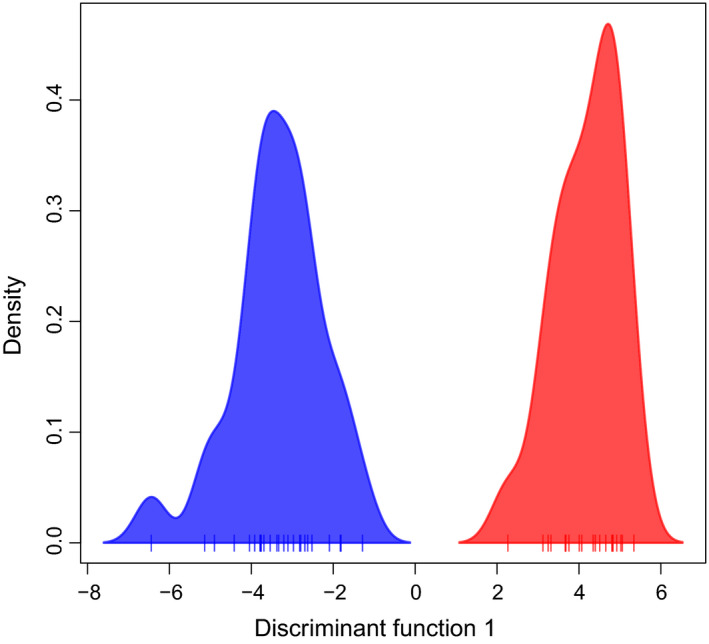
Density plot of domestic carp specimens along the first discriminant function from Discriminant Analysis of Principal Components (DAPC). Two domestic strain groups are shown using different colors: blue for Northern strains and red color for the European group

We also conducted discriminant analyses of the principal component between Ponto–Caspian (European) and Northern strain groups. We combined Angelinskii, Cherepet, Ropsha, Stavropol, and Ukrainian strains in one group, and the remaining domestic separately to explore strain group differences. The sample density along the discriminant function clearly separates these two groups of domestic common carp strains (Figure 4).

**Figure 5:**
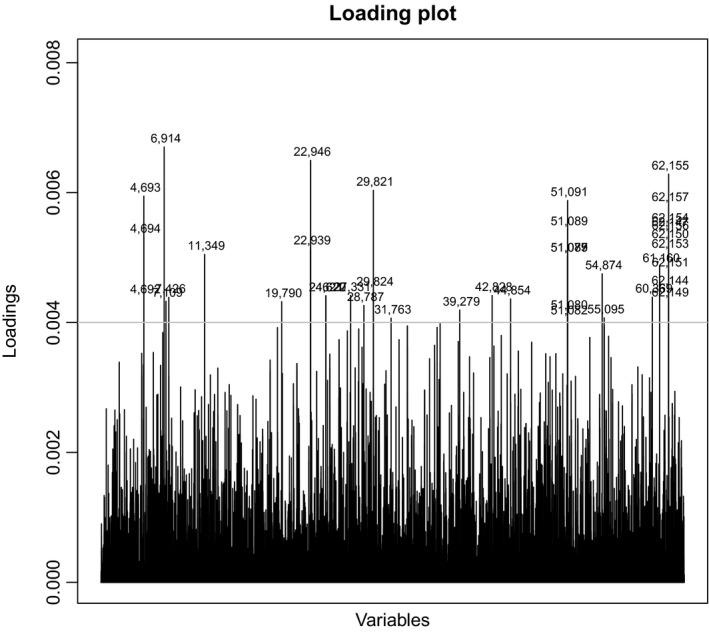
Loading plot contribution of alleles into differentiation of common carp domestic strain groups

However, the discrimination power of each explored locus was very low, despite a significant number of the differentiating loci. We found that only eight alleles exceed the discriminating power of 0.4% (Figure 5), while its mean value was approximately 0.1%. Nevertheless, the high number of such loci enables discrimination of all the strains in the analyzed groups with great statistical support.

**Figure 6:**
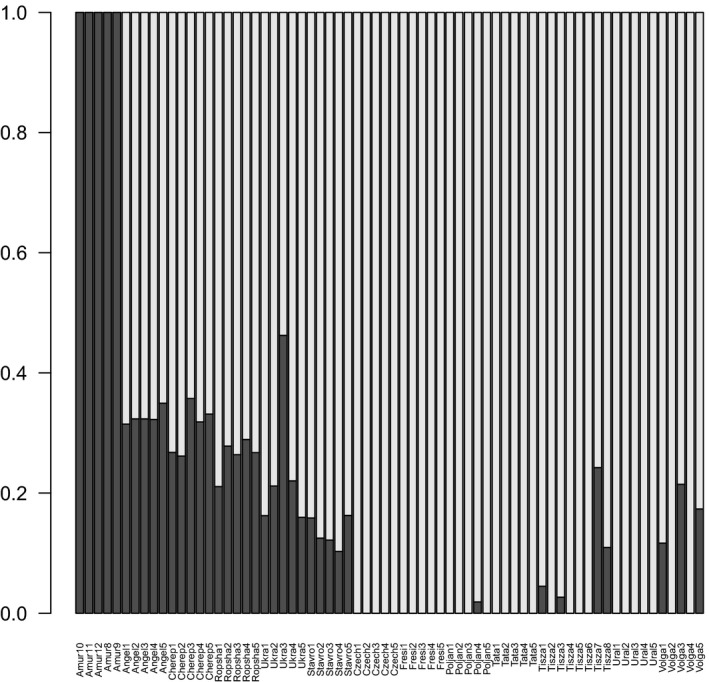
NGSAdmix analysis of the European and Northern domestic carp strains and wild carp populations. Dark gray color specifies Amur carp heredity contribution in each specimen

To estimate the genetic contribution of wild Amur carp into domestic strains, we conducted admixture analyses (Figure 6), which showed that the genetic contribution of Amur carp into Northern common carp domestic strains is much higher than to Ponto–Caspian (European) strains. Moreover, a few specimens from Ponto–Caspian (European) common carps, especially from Tisza and Volga wild populations, also have a notable wild Amur carp genetic contribution.

A total of 2,618 SNP loci (with *p‐*value <.05) with different allele frequency between strains were blasted to zebrafish (*Danio rerio*) peptide database, and 724 zebrafish genes were found to contain the SNPs which distinguished European and Northern common carp strain groups. *D. rerio* gene IDs were then converted into universal gene names for the functional analysis.

Analysis of gene categories, overrepresented in European and Northern common carp discrimination gene set, revealed an excess of genes responsible for the motor molecular activity, hydrolase activity, and GTPase binding. In particular, the zebrafish microtubule‐binding gene category (GO:0008017) contains 173 genes, and 14 of them were found in our discrimination gene set (*p‐*value = .000335); the zebrafish tubulin‐binding category (GO:0015631) has 196 genes in total, and 15 of them were present in our dataset (*p‐*value = .000363); the zebrafish cytoskeletal protein‐binding category (GO:0008092) comprises 493 genes in total, and 27 genes were described in our set (*p‐*value = .000577).

## 4 DISCUSSION

The common carp domestication is a fascinating history of breeding and selection of dozens domestic strains with different zootechnical characteristics around the world. Previously, it has been shown that domestic common carp strains have been divided into two distinct groups. Asian strains include Oujiang, Hebao, Xingguo and Koi, and European (Ponto–Caspian) strains (Xu et al., 2014).

Here, we describe a new *C. carpio* strain group, named Northern carp strain group, which includes strains mostly created in former Soviet Union starting from the 1930s (Kirpitchnikov & Balkashina, 1935). We suppose the essential point of these strains development was a breeding program based on the wild Amur carp (C*. carpio haematopterus*) that gave for the Northern strains several traits related to cold tolerance.

The origin of the “Northern group strains” is not well‐documented, making molecular confirmation of this breeding extremely important step in future breeding programs. Moreover, the accurate identification of *C. carpio* strains should be a priority to increase the production efficiency and sustainability of their production.

It is known that the Ropsha strain was created by direct crossing with wild Amur carp, while the Ukrainian strain was created by breeding to Ropsha hybrids and the Angelinskii strain originated from breeding Ukrainian carp strain females and Ropsha carp strain males (Bogeruk, 2004). However, admixture with wild Amur carp is not shown in the pedigree records of many other Northern group strains. In particular, the Stavropol strain origin is attributed to crossing a local wild carp (Stavropol Kray, Southern Russia) with the Tata strain–Hungarian strain (Bogeruk, 2004), which are not descendants of wild Amur carp. Our genomic data shed light into the puzzling origin of the Stavropol carp strain, which turned out to have wild Amur genetic introgression.

The Ropsha domestic strain is also distant from wild Amur carp (see Figure 2), despite the fact that it was formed by crossbreeding European strains with the latter. This could mean that the Ropsha strain traits were formed not only by Amur carp alleles admixture but also by artificial selection to low‐temperature resistance, and different allele combinations were fixed in the domestic strain.

A few studies have previously described specific traits of the Northern strains and their differences from European domestic carp strains (Ludannyĭ et al., 2006, 2010), but they did not assume the impact of the Amur strain on the most Northern strains. We further demonstrated for the first time that the wild Amur common carp ancestry has impact on Northern strains, but almost not on the European strains. Moreover, we demonstrated that the strain groups are genetically distinct. But whether the distinction is consequence of only Amur admixtion or it is also result of different vectors of selection remains open to question.

Due to the carp genome tetraploidy, there is a possibility that part of the loci would be mix‐mapped because of undistinguishable paralogous sequences, which can cause an excess of heterozygosity in loci in the analysis. The Hardy–Weinberg test has shown that only 5% loci (3,618 from the 65,686) have a deviation from the equilibrium. That means that paralogous sequences in the common carp genome mostly are well distinguished from each other, likely due to the allotetraploidization nature of the *C. carpio* genome.

Establishing a connection between genotypes and traits is the main goal of a genetic investigation, but the underlying mechanisms of implementation of genetic information remain quite unclear. To determine the genetic impact on a trait of interest, the common way is to compare different groups by allele frequencies and identify genes with different allele frequencies between groups.

In an effort to explain the genetic mechanisms of cold resistance, we have identified genes that differ in allele frequency between European and Northern domestic carp strains. We found 724 such genes, which belong to different categories. The most represented genes have molecular functions such as molecular motor activity, hydrolase activity, and GTPase binding. There are a number of reports of cold tolerance genes in fish (Kirpitchnikov & Balkashina, 1935). In most cases, these genes are defined by having varying expression levels in different temperature conditions.

While comparing our gene list with cold tolerance genes in the literature, we found some common genes and gene categories. For example, from 12 GO categories enriched in our gene list, two categories (GO:0005524—ATP binding, *p‐*value: .0003 and GO:0000166—nucleotide binding, *p‐*value: .0084) match to GO categories, defined as cold stress response in pufferfish—*Takifugu fasciatus* (Wen et al., 2019). Second of the categories (GO:0000166—nucleotide binding) also mentioned as cold responsive in Amur carp, defined by transcriptome analyses (Liang, Chang, He, & Tang, 2015). Unfortunately, the appropriate gene IDs in those publications are absent, which makes any comparison between gene sets impossible.

In another study investigating transcriptome changes in blue tilapia (*Oreochromis aureus*) exposed to low temperature (Nitzan et al., 2019), only 6 out of 312 genes up‐regulated with cold coincided with our gene set. Only one from 170 down‐regulated genes was present in our common carp enriched gene set. We found that there were three GO categories (mentioned above and 0021551—central nervous system morphogenesis) enriched in both blue tilapia exposed to cold (Nitzan et al., 2019) and common carp selected for cold tolerance. However, there were no coinciding GO categories in the down‐regulated gene set.

The identification of common genes with different allele frequencies in our study and differential expression with cold suggests that the cold tolerance mechanisms may be partly the same. However, genetic and epigenetic changes during adaptation do not have to be necessarily the same; for example, epigenetic regulatory changes can have a complementary action to compensate genetic variations (Artemov et al., 2017). Further investigations are required to comprehend the genetic mechanisms underlying trait formation and its possible hidden patterns of inheritance.

Our results provide insights into the genomic variability, population structure, and admixture events that occurred during domestication of common carp strains. In the work, we obtained molecular evidence, allowing to trace origin of Russian carp strains without any assistance of reference data as breeding card or schemes of interbreed crossing. It is an important statement, because this shows that the strains are much more genetically integrated to each other than previously thought. It is worth noting that not only strains that are cultivating in northern regions have Amur genome introgression, but also southern strains, which originated in the warm environment of Southern Russia, have traces of Amur genome. Moreover, our genomic toolbox forms the basis to develop a high‐density SNP array for accurate discrimination between common carp strains, which will be useful to identify escapees from aquaculture farms and to quantify introgression in wild populations.

## CONFLICT OF INTEREST

The authors declare no conflict of interest.

## AUTHOR CONTRIBUTION

**Artem V. Nedoluzhko:** Writing‐original draft (equal). **Natalia V. Slobodova:** Investigation (supporting); Methodology (supporting). **Fedor Sharko:** Software (lead); Visualization (supporting). **Gulmira M. Shalgimbayeva:** Data curation (equal); Resources (equal). **Svetlana V. Tsygankova:** Investigation (equal). **Eugenia S. Boulygina:** Formal analysis (equal). **Zsigmond Jeney:** Data curation (equal). **Van Q. Nguyen:** Writing‐review & editing (lead). **Đức T. Nguyen:** Resources (equal). **Thế T. Pham:** Resources (equal). **Alexander A. Volkov:** Conceptualization (equal); Data curation (equal). **Jorge M. O. Fernandes:** Writing‐original draft (equal); Writing‐review & editing (equal). **Sergey M. Rastorguev:** Conceptualization (equal); Supervision (equal); Visualization (equal); Writing‐original draft (equal); Writing‐review & editing (equal).

## Data Availability

All DNA sequences have been uploaded to the NCBI Sequence Read Archive; BioProject ID PRJNA573845; sample IDs are specified in Table 1.
